# Effectiveness of ultraviolet-C disinfection systems for reduction of multi-drug resistant organism infections in healthcare settings: A systematic review and meta-analysis

**DOI:** 10.1017/S0950268823001371

**Published:** 2023-08-30

**Authors:** YanLin Sun, Qi Wu, Jinzhi Liu, Qian Wang

**Affiliations:** 1 Day Surgery Center, The Affiliated Qingdao Central Hospital of Qingdao University, Qingdao, China; 2 Department of Infection Management, The Affiliated Qingdao Central Hospital of Qingdao University, Qingdao, China; 3 Department of Gastroenterology, The Affiliated Qingdao Central Hospital of Qingdao University, Qingdao, China

**Keywords:** healthcare-associated infection, HCAI, meta-analysis, multi-drug-resistant organisms’, systematic review, ultraviolet C

## Abstract

This study aimed to summarise the findings of the studies assessing the effectiveness of ultraviolet C (UV-C) room disinfection in reducing the incidence rate of healthcare-associated multi-drug-resistant organism (MDRO) infections. A systematic screening was conducted using PubMed, EMBASE, and Scopus for randomised controlled trials (RCTs), quasi-experimental studies, and before–after studies, which assessed the efficacy of the UV-C disinfectant system in reducing the incidence of MDRO infections. A random-effects model was used for the analysis. Effect sizes were described as incidence rate ratio (IRR) with 95% confidence intervals (CI). Nine studies were included, all of which were conducted in the USA. No statistically significant reduction in *Clostridioides difficile* (CD) (IRR: 0.90, 95% CI; 0.62–1.32) and vancomycin-resistant enterococcal (VRE) infection rates (IRR 0.72, 95% CI; 0.38–1.37) was observed with the use of UV-C, but the risk of Gram-negative rod infection was reduced (IRR 0.82, 95% CI; 0.68–0.99).

## Introduction

Nosocomial infections, also called healthcare-associated infections (HCAIs), are reported to account for approximately 7% of all infections in developed countries and 10% in developing countries [1, 2]. Recent evidence suggests that nosocomial infections affect nearly 15% of all hospitalised patients [3] and are associated with prolonged hospital stay, significant disability, and economic burden. Studies conducted in high-income settings in the USA and Europe showed that the incidence density of such infections is around 13–20 episodes per 1000 patient days [4] and is associated with a high financial burden [5]. This burden is expected to be much higher with the increasing emergence of multi-drug-resistant organisms (MDROs) [5]. Recent studies have shown that >70% of the bacteria implicated in HCAI are usually resistant to one or more of the antimicrobials used for the initial treatment of patients [6], and the attributable cost increase in treating resistant organisms ranges from 4000 to 4500 USD per infection per patient [7, 8].

Multi-drug-resistant bacteria can survive in the hospital environment for long periods [9], and all surfaces, porous or non-porous, in patient’s rooms are highly susceptible to contamination [10]. Consequently, effective infection prevention programmes have environmental hygiene as an integral component. A wide range of chemical disinfectants are commonly used in healthcare settings and include surface disinfectants such as quaternary ammonium compounds, sodium hypochlorite, peracetic acid, and liquid hydrogen peroxide [10]. No-touch technologies in addition to conventional cleaning measures are commonly used in hospital settings and include exposure to ultraviolet light or hydrogen peroxide vapour or mist [11–13]. Ultraviolet light sources are broadly categorised as UV-C devices and those that utilise pulsed xenon–UV light (PX-UVL). The former consist of mercury bulbs that emit continuous radiation of wavelength ranging from 200 to 270 nm [14, 15], while a PX-UVL system is characterised by short high-intensity bursts of radiation of UV wavelengths (100–280 nm) and visible (380–700 nm) spectra [14, 15].

A meta-analysis by Dong et al. [16] has shown that PX-UV may be useful in reducing the incidence rate of infections with *Clostridium difficile* (CD) and methicillin-resistant *Staphylococcus aureus* (MRSA) but was not effective in reducing the rates of vancomycin-resistant enterococcal (VRE) infection. Likewise, Marra et al. [17] pooled data on both types of UV technologies and found a statistically significant decrease in both CDI and VRE infection rates, but rates of MRSA and Gram-negative MDROs were unaffected. However, the latter analysis included only two studies utilising UV-C.

Recently, several studies have examined the effect of UV-C disinfection system on the rates of MDRO infections in hospitals. The current review aimed at synthesising the findings of all studies assessing the impact of UV-C room disinfection on reducing HCAI infection rates. In particular, the outcomes were related to the effect of UV-C disinfection on the risk of CDI, VRE, and Gram-negative multi-drug-resistant pathogens. The decision to focus only on UV-C disinfection systems (and exclude PX-UVL studies) was driven by the specific research question and objectives of the meta-analysis. The primary focus of our analysis was on disinfection systems that utilise continuous-wave UV-C light. PX-UVL systems differ from the latter in terms of the light source, technology, and application protocols. Nevertheless, the methods aim to achieve disinfection by damaging the genetic material of microorganisms. We considered that these differences made it challenging to directly compare the outcomes of PX-UVL evaluations with conventional UV-C systems.

## Methods

### Selection of studies

The review protocol was registered at PROSPERO (registration number CRD42023405885). Given its nature, oversight by an institutional board was not required before registration. The search strategy is presented in Supplementary Table S1. Three databases, that is PubMed, EMBASE, and Scopus, were screened for English language studies published up to 15 February 2023, in accordance with Preferred Reporting Items for Systematic Reviews and Meta-Analyses (PRISMA) [18]. The inclusion criteria were as follows: studies that had assessed the efficacy of an ultraviolet C (UV-C) disinfectant system for reduction in the incidence of MDRO infections; were conducted in all healthcare settings; of all sample sizes and types of patient population; and randomised controlled trials (RCTs), quasi-experimental studies, and before–after interventions. The exclusion criteria were as follows: those assessing the effect of PX-UVL; evaluations of the efficacy of UV-C in combination with other infection control measures; and reviews, case reports, and case series. After the removal of duplicates, the titles and abstracts of studies were independently screened by two of the study investigators for potential inclusion. The full texts of the remaining studies were then read, and final decisions for inclusion were made. A senior author was consulted in case of any discrepancies.

### Data extraction and statistical analysis

Data were extracted using a pretested electronic template that consisted of variables related to study identifiers (author’s name, year of publication, study design, and country of study), type of health facility where the study was conducted, duration, and findings of relevance. Statistical analysis was conducted using STATA 16 software (Texas, USA). The pooled effect sizes were reported as an incidence rate ratio (IRR) with 95% confidence intervals (CI). We decided, *a priori*, to use a random-effects model for all analyses to account for potential variability such as characteristics of the hospitals studied, study design, and method of data collection. It was assumed that these differences would have led to substantial heterogeneity in the reported findings. A revised Cochrane risk-of-bias tool for randomised trials (RoB 2) was used [19]. For ‘before–after’ studies, the quality assessment tool developed by the National Heart Lung and Blood Institute [20] was used, and Egger’s test and funnel plots were used for the assessment of publication bias [21]. P < 0.05 was statistically significant. A subgroup analysis was conducted based on the study design (i.e. RCT/quasi-experimental and before–after design), for the risk of CDI as an outcome.

## Results

**Figure 1.**
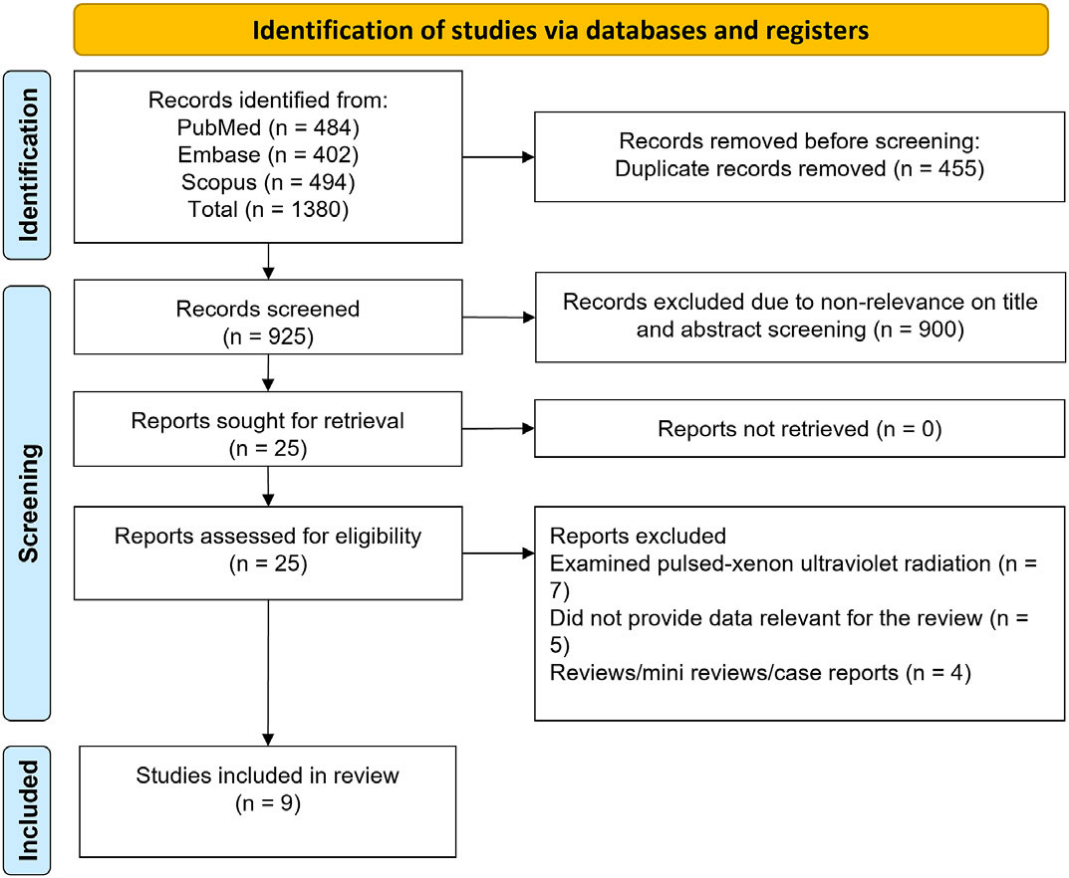
Selection process of studies included in the review.

A systematic search across three databases identified 1380 studies. After the removal of the 455 duplicates, 925 unique studies remained. Screening based on their title and abstract led to the further exclusion of 900 studies. The full texts of the resulting 25 studies were screened, and an additional 16 were excluded (Figure 1) leaving nine studies [22–30] for this meta-analysis (summarised in Table 1). All studies were conducted in the USA and five were of a ‘before–after’ design, two were classed as quasi-experimental, and two were of a cluster-randomised crossover design. The included studies examined mainly CD, VRE, Gram-negative rod infection, and MRSA. Two studies specifically studied Gram-negative MDROs including *Klebsiella*, *Acinetobacter*, *Pseudomonas,* and *E. coli.* Schaffzin et al. [30] defined a Gram-negative as an isolate that was non-susceptible (intermediate or resistant) to at least one agent in at least three of nine antibiotic classes (anti-pseudomonal penicillin, third- or fourth-generation cephalosporin, carbapenem, fluoroquinolone, aminoglycoside, penicillin and beta-lactamase inhibitor, monobactam, polymyxin, and folate inhibitor). Five of the studies were conducted in academic medical facility or tertiary care hospitals, two in community hospitals, and one each in Veteran’s Health Administration (VHA) hospitals, and in a tertiary or community or VHA setting. The quality assessments for the cluster-randomised and quasi-experimental studies and for ‘before–after’ design studies are presented in Supplementary Figure S1 and Supplementary Table S2, respectively. It was concluded that most studies had a moderate risk of bias.

**Table 1.** Characteristics of the studies included in the meta-analysis

References	Study design and country	Hospital type (No. of beds)	Study period and comparisons	Outcomes
Rock [22]	Cluster-randomised crossover trial USA	Academic medical facility (1059) Cancer and solid organ transplant in-patient units	Phase 1: 12 months and 15 days Washout: 5 weeks Phase 2: 12 months and 15 days UV-C + standard environmental cleaning versus standard environmental cleaning	Vancomycin-resistant enterococcal infection (VRE): IRR 0.98 (95% CI; 0.78, 1.22) Clostridioides difficile (CD) infection: IRR 1.43 (95% CI; 0.93, 2.21)
McMullen [23]	Before–after USA	Acute care community hospital (472)	Pre-intervention: 12 months Post-intervention: 21 months Pre-intervention: Standard daily manual disinfection protocol Intervention: manual disinfection protocol along with UV-C-based disinfection	CD infection: IRR 1.34 (95% CI; 0.84, 2.15)
Anderson [24]	Cluster-randomised crossover trial USA	Nine hospitals representing multiple types (tertiary, community, Veterans Affairs)	Each strategy used at every study hospital for four consecutive 7-month study periods. Each study period consisted of a 1-month wash-in period followed by a 6-month period of data collection Three strategies for enhanced terminal disinfection tested against the standard terminal disinfection *Comparison of interest for this review:* UV-C + standard quaternary ammonium disinfectant (bleach for *C. difficile*) versus standard quaternary ammonium disinfectant (bleach for *C. difficile*)	VRE infection: IRR 0.46 (95% CI; 0.26, 0.82) CD infection: IRR 0.96 (95% CI; 0.61, 1.52) Methicillin-resistant staphylococcal infection (MRSA): IRR 0.73 (95% CI; 0.51, 1.04)
Abosi [25]	Before–after USA	Academic medical centre (811)	9 months UV-C + sporicidal agent (bleach wipes) versus sporicidal agent (bleach wipes)	CD infection: IRR 1.05 (95% CI; 0.70, 1.58)
Steele [26]	Before–after USA	Academic children hospital (364) Paediatric haematology–oncology unit	Pre-intervention: 42 months Post-intervention: 18 months Pre-intervention: manual disinfection protocol along with UV-C-based disinfection. Post-intervention: standard daily manual disinfection protocol	CD infection: IRR 0.38 (95% CI; 0.24, 0.61)
Napolitano [27]	Before–after USA	Community hospital (420) All patient rooms, hospital-wide	6 months Continuously monitored and frequently UV-C treatment; incidence of infection before and after the intervention period compared	CD infection: IRR 0.54 (95% CI; 0.03, 10.58) VRE infection: IRR 0.88 (95% CI; 0.05, 15.36)
Pegues [28]	Quasi-experimental with interrupted time series USA	Academic tertiary care hospital (789) Haematology–oncology units	Pre-intervention: 12 months Post-intervention: 12 months Incidence rates of *C. difficile* infection compared between the baseline and intervention period	CD infection: IRR 0.75 (95% CI; 0.43, 1.30)
Goto [29]	Quasi-experimental USA	Veterans’ Health Administration (VHA) hospitals	Pre-intervention: standard disinfection protocol Intervention: enhanced terminal room cleaning with ultraviolet C (UV-C)	Hospital-onset Gram-negative rod infection: IRR 0.81 (95% CI: 0.66, 0.97)
Schaffzin [30]	Before–after USA	Large paediatric referral facility (449)	Pre-intervention: standard disinfection protocol (hydrogen peroxide or bleach) Intervention: enhanced cleaning with ultraviolet C (UV-C)	Hospital-onset Gram-negative rod infection: IRR 2.12 (95% CI: 0.32, 14.2)

**Figure 2.**
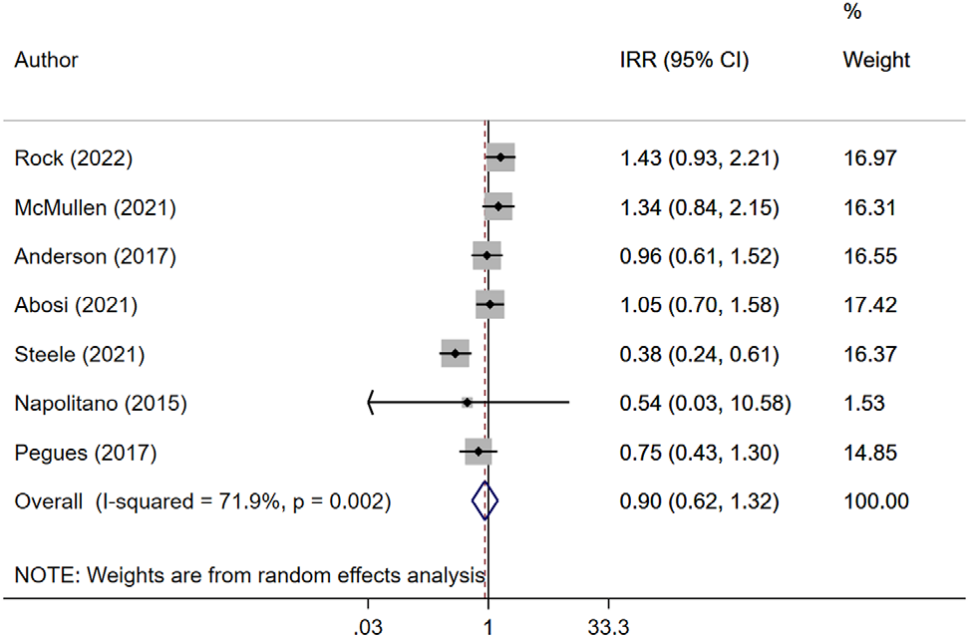
Forest plot of IRRs of *Clostridioides difficile* (CD) infection for UV-C versus control.

**Figure 3.**
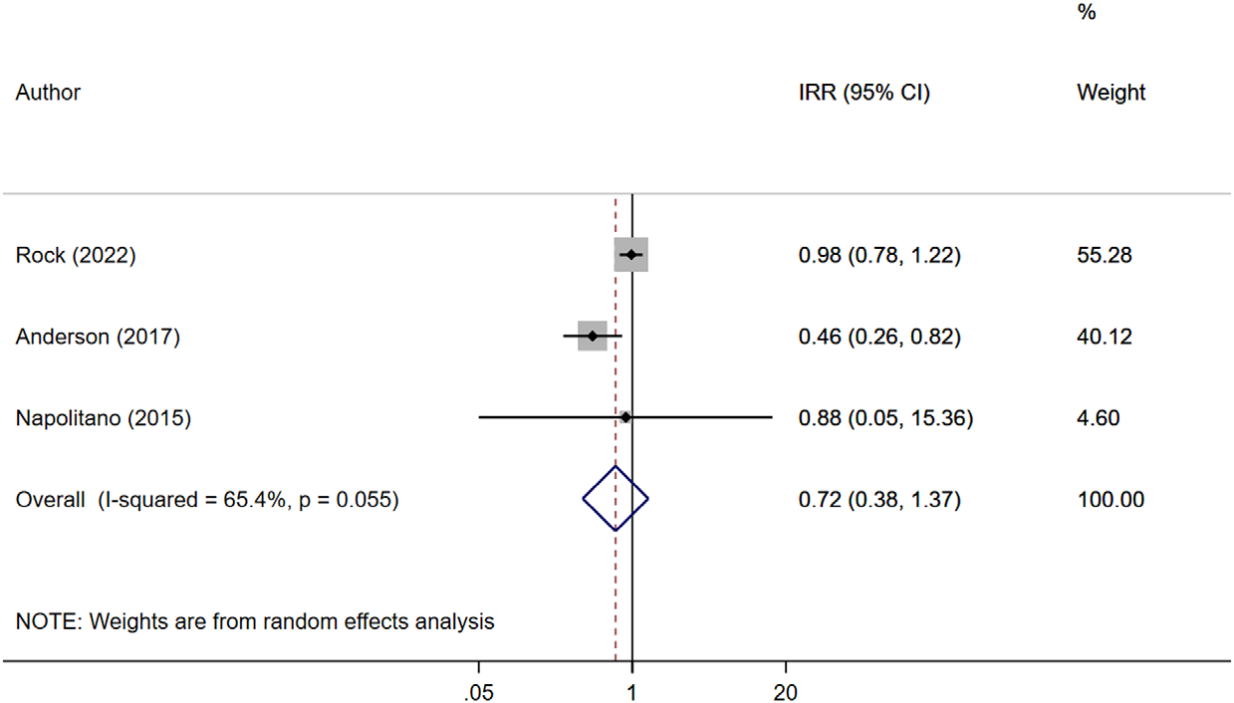
Forest plot of IRRs of vancomycin-resistant enterococcal infection (VRE) for UV-C versus control.

**Figure 4.**
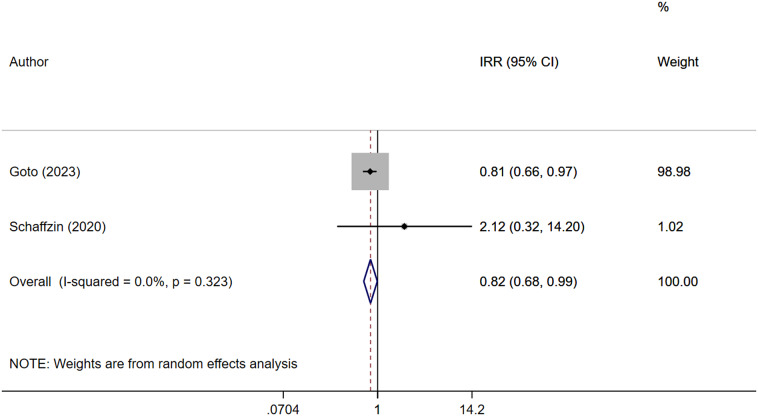
Forest plot of IRRs of Gram-negative rod infection for UV-C versus control.

Pooled analysis indicated no statistically significant reduction in CD infection rates with the use of ultraviolet C disinfection systems (IRR: 0.90, 95% CI; 0.62–1.32, I^2^ = 71.9%, *N* = 7) (Figure 2). Subgroup analysis based on the study design also showed no effect of this system on risk of CD infection for both RCT/quasi-experimental studies (IRR: 1.04, 95% CI; 0.72–1.50, I^2^ = 43.2%, *N* = 3) and ‘before–after’ design (IRR: 0.80, 95% CI; 0.40–1.59, I^2^ = 81.4%, *N* = 4) (Supplementary Figure S2). No publication bias was shown by Egger’s test (*P* = 0.728) and funnel plots (Supplementary Figure S3). Similarly, no significant reduction in VRE infection was observed (IRR 0.72, 95% CI; 0.38–1.37, I^2^ = 65.4%, *N* = 3) (Figure 3), and no evidence of publication bias was found (Supplementary Figure S4). UV-C systems appeared to reduce the risk of Gram-negative rod infection (IRR 0.82, 95% CI; 0.68–0.99, I^2^ = 0.0%, *N* = 2), but the number of studies reporting this outcome was small (Figure 4). No evidence of publication bias was found (Supplementary Figure S5).

## Discussion

Our study did not find any evidence of the benefit of using UV-C-based disinfectant systems in healthcare facilities to reduce the incidence of nosocomial infections, particularly MDROs such as CD and VRE. Nevertheless, some studies showed that UV-C (wavelength 200–270 nm) is effective in reducing the risk of Gram-negative rod infections by inducing DNA and RNA damage, through dimerisation of pyrimidine molecules, thereby reducing the replication of microorganisms [31, 32]. UV-C at the wavelengths of 250–270 nm appears to be the most efficient due to its maximal absorption by microbial nucleic acids [33]. However, one of the disadvantages of UV-C is that its penetration is affected by the presence of organic matter [34]. Additionally, there is an issue of costs and the requirement for the training of the personnel. We found no advantages to the use of UV-C in healthcare settings as an adjunct to conventional infection prevention modalities to reduce the incidence of MDRO.

The aspect of cost-effectiveness and staff training should also be considered further. Two previous studies that have conducted cost-effectiveness evaluation of ultraviolet disinfection systems after terminal cleaning [28, 35] have shown the cost to be around 200,000 to 300,000 USD per year. However, on average, cases of CDI and VRE can lead to a cost of *c.* 14,000 USD/case [36, 37]. Nursing professionals are essential for implementing disinfection protocols in healthcare facilities, as they are directly involved in the cleaning and disinfection of shared medical monitoring devices. It is therefore critical that any advancement in technology be known to them. In this regard, the current review further emphasises the importance of ‘no-touch’ disinfection systems in health facilities.

With the assumption that, in the included studies, the dosing and duration of exposure were appropriate, there may be some possible explanations for the lack of effectiveness of UV-C disinfectant systems. One reason could be the use of disinfectant as part of the standard protocol offered in both study groups. High compliance with disinfectants such as bleach or standard quaternary ammonium compounds may have led to relatively few residual spores for the UV-C device to eliminate. Second, UV-C disinfection relies on direct line-of-sight exposure to effectively kill microorganisms, and inadequately exposed surfaces to UV-C light could result in incomplete disinfection. Recent findings suggest that the role of the environment in the transmission of *C. difficile* may not be as significant as previously believed. Eyre et al. [38] examined 1250 isolates from cases of symptomatic CDI over four years using whole-genome sequencing. Surprisingly, possible environmental contamination was found to be responsible for linking only 2% of patients with genetically related *C. difficile* isolates. Furthermore, differences in study design, protocols, equipment, or the specific UV-C system most likely contribute to variations in effectiveness, as well as room size and layout, UV-C system placement, and operator training, which can influence the overall performance and outcomes of studies. On the contrary, Steele et al. [26] showed that UV-C irradiation reduced the risk of CDI in a paediatric haematology–oncology unit and suggested that this might depend on the pre-intervention CDI burden and that such high-risk units could have maximum benefit from use of such systems.

There are some limitations to our study. First, all reviewed studies were conducted in the USA, that is a high-income setting, and therefore, the generalisability of the findings is limited. Second, most of the included studies had a ‘before–after’ design, compared current data with a historical control group, and therefore did not consider changes in hospital practices that occur with time. Third, the included studies varied in terms of intervention sites and standard methods of disinfection used. For instance, some studies were conducted in cancer and solid organ transplant in-patient units where patients would have lower immunity and thus be at an increased risk of HCAI. Finally, our approach of limiting the study scope to English language studies could have introduced a degree of bias through missing relevant studies, as well as geographic, cultural, and publication bias.

## Conclusion

The use of UV-C had no measurable impact on the incidence of CD and VRE infections but might be of some advantage in reducing the risk of Gram-negative rod infection, albeit with low confidence. Further studies to support or refute the outcome of this meta-analysis are needed.

## Supporting information

Sun et al. supplementary material 1 Sun et al. supplementary material

Sun et al. supplementary material 2 Sun et al. supplementary material

Sun et al. supplementary material 3 Sun et al. supplementary material

Sun et al. supplementary material 4 Sun et al. supplementary material

Sun et al. supplementary material 5 Sun et al. supplementary material

Sun et al. supplementary material 6 Sun et al. supplementary material

Sun et al. supplementary material 7 Sun et al. supplementary material

## Data Availability

The authors confirm that the data supporting the findings of this study are available within the article and its supplementary materials.

## Supplementary material

For supplementary material accompanying this paper visit http://doi.org/10.1017/S0950268823001371. click here to view supplementary material
