# Supplementary figures and images for: Effectiveness of ultraviolet-C disinfection systems for reduction of multi-drug resistant organism infections in healthcare settings: A systematic review and meta-analysis
Source: Epidemiol Infect. 2023 Aug 30;151:e149. doi: 10.1017/S0950268823001371 (PMC10540170; doi:10.1017/S0950268823001371)

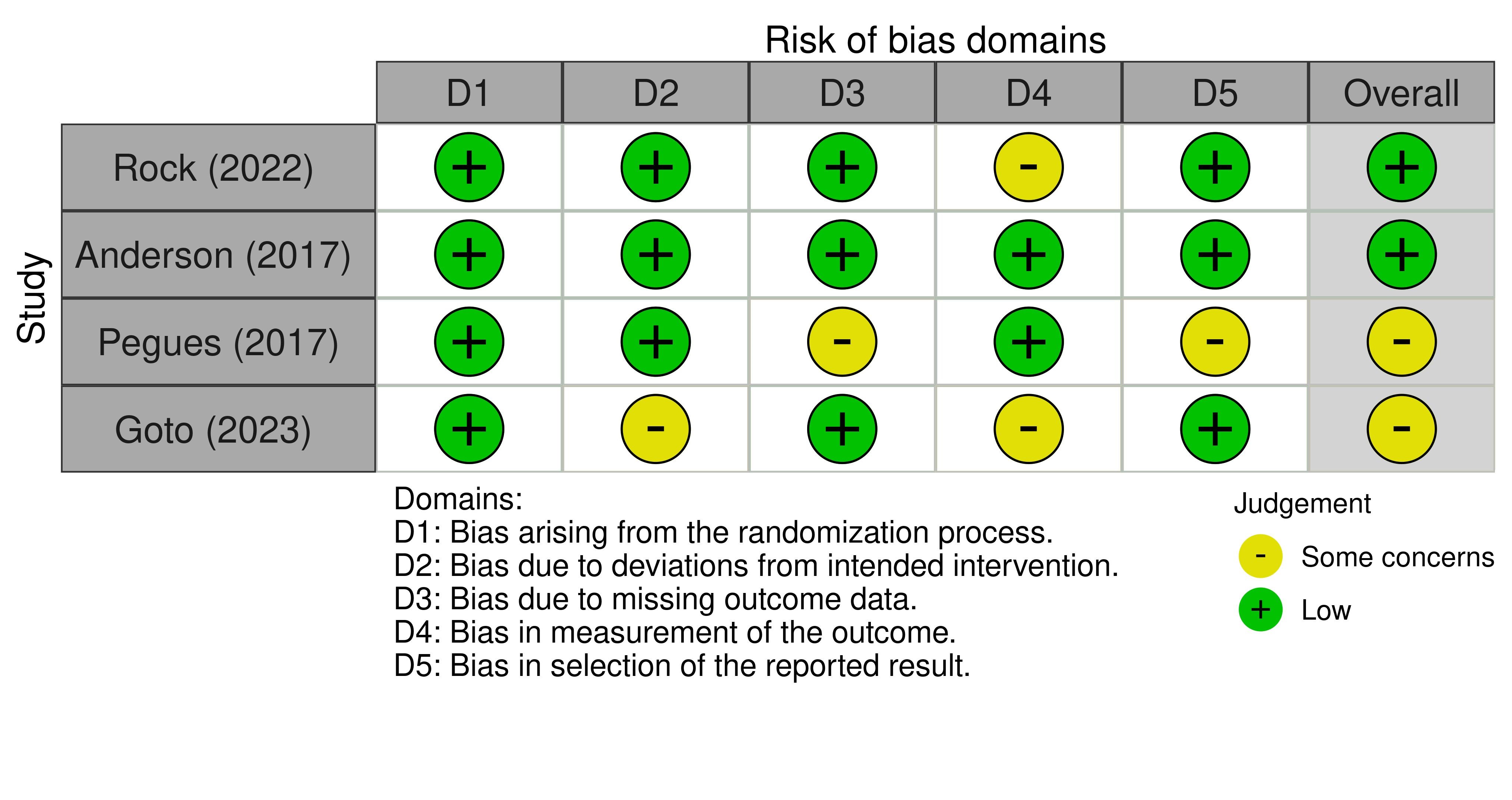

Supplement: Sun et al. supplementary material 1 — Sun et al. supplementary material [file S0950268823001371sup001.jpg]

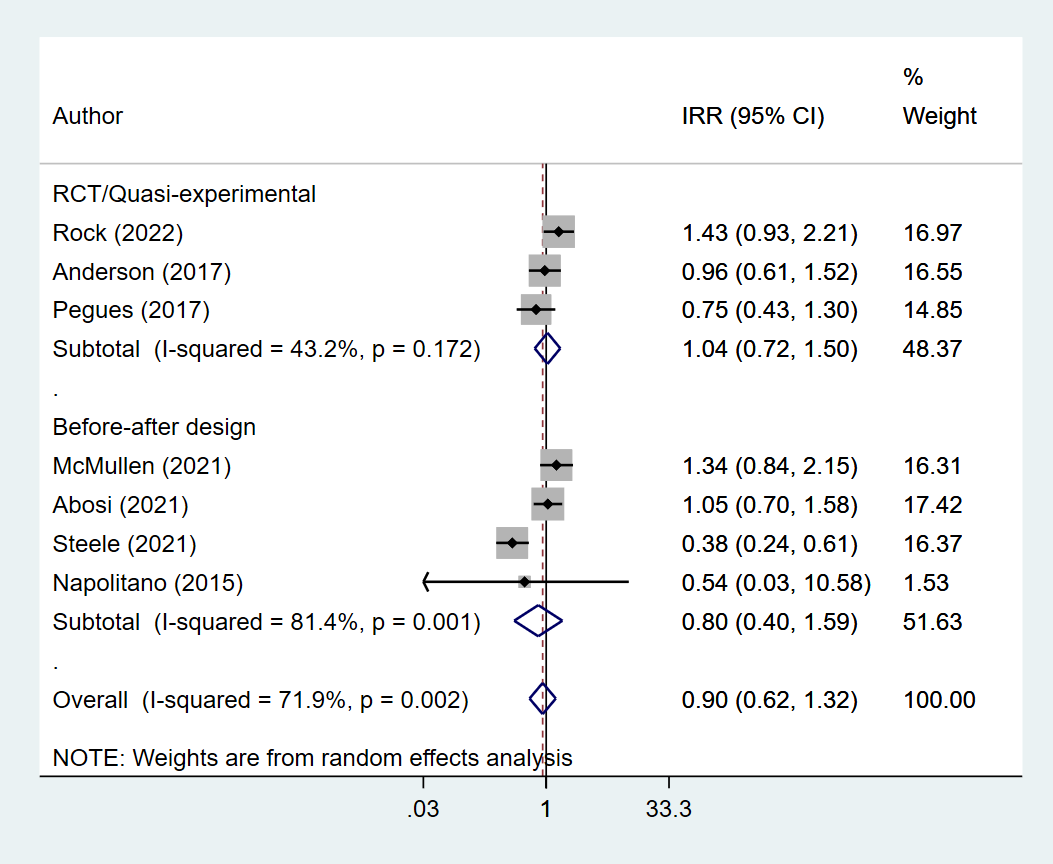

Supplement: Sun et al. supplementary material 2 — Sun et al. supplementary material [file S0950268823001371sup002.tif]

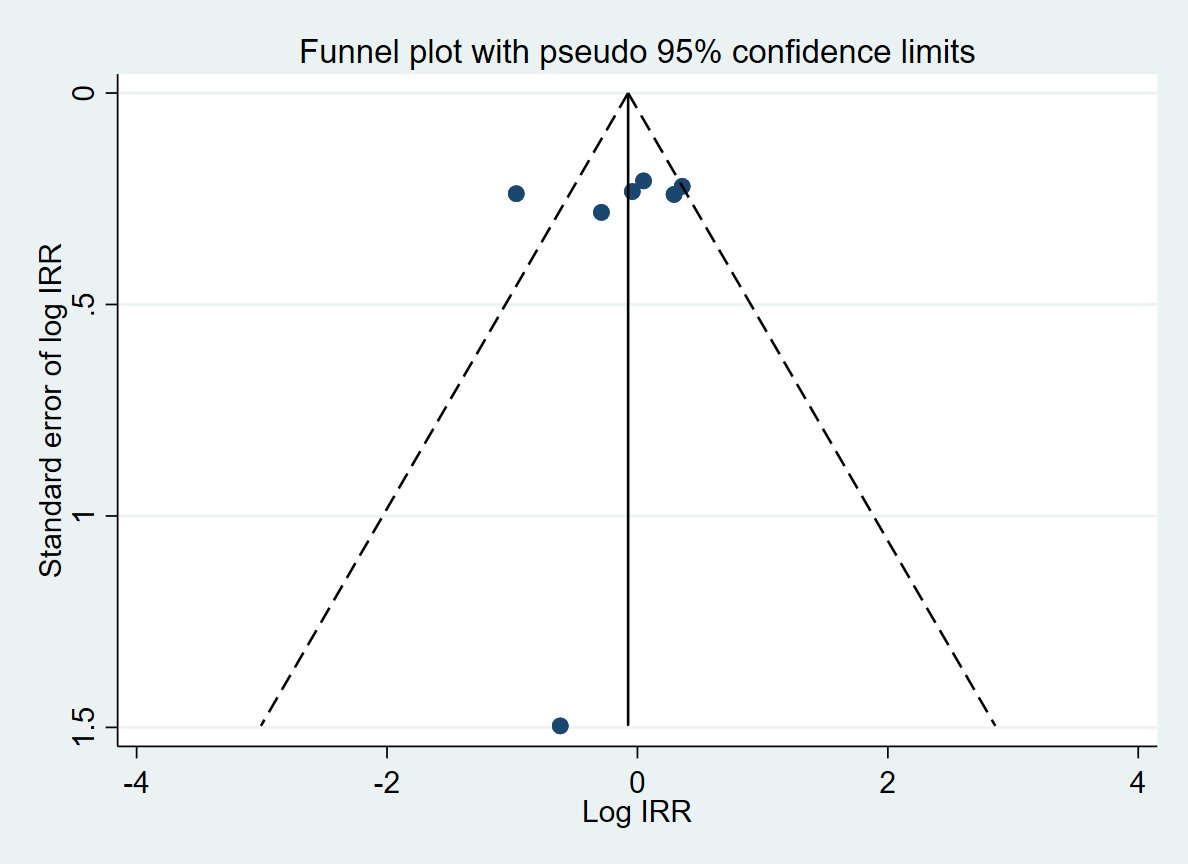

Supplement: Sun et al. supplementary material 3 — Sun et al. supplementary material [file S0950268823001371sup003.tif]

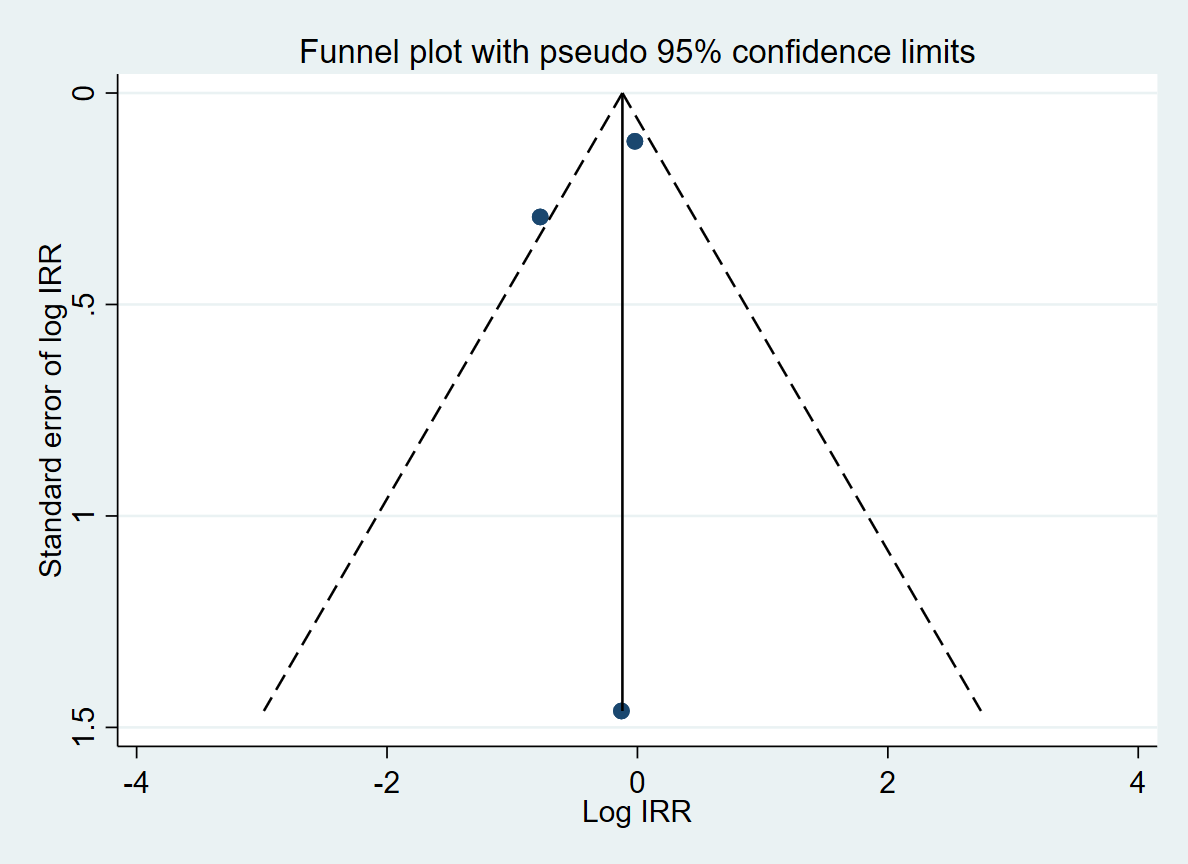

Supplement: Sun et al. supplementary material 4 — Sun et al. supplementary material [file S0950268823001371sup004.tif]

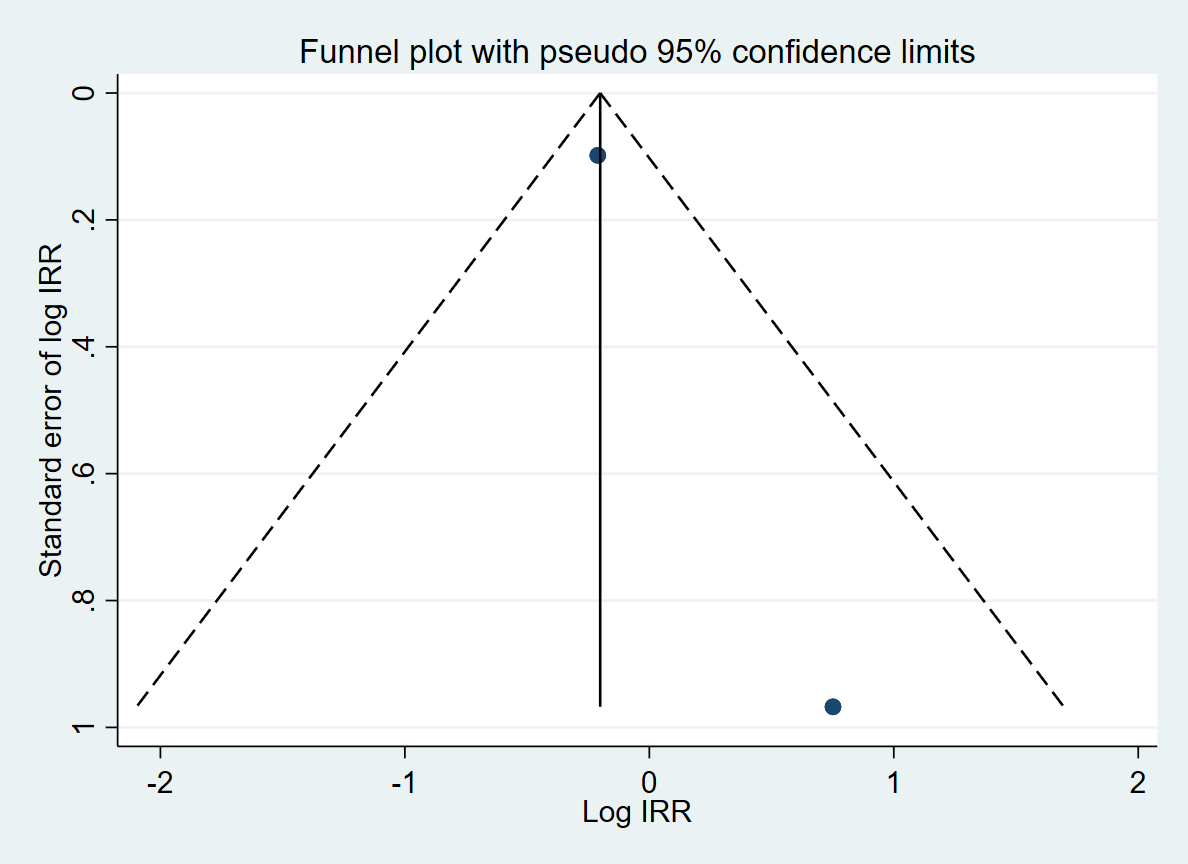

Supplement: Sun et al. supplementary material 5 — Sun et al. supplementary material [file S0950268823001371sup005.tif]
