# Supplementary material for: Effectiveness of ultraviolet-C disinfection systems for reduction of multi-drug resistant organism infections in healthcare settings: A systematic review and meta-analysis
Source: Epidemiol Infect. 2023 Aug 30;151:e149. doi: 10.1017/S0950268823001371 (PMC10540170; doi:10.1017/S0950268823001371)
Supplement: Sun et al. supplementary material 6 — Sun et al. supplementary material [file S0950268823001371sup006.docx]

**Supplementary table 1. Search strategy used to identify studies**

1. **Search strategy for PubMed**

#1 (ultraviolet-C OR PX-UVL OR pulsed xenon ultraviolet OR disinfection OR UV light)

#2 (nosocomial infection OR health care infection OR clostridium difficile OR vancomycin resistant OR enterococci OR MRSA OR methicillin resistant staphylococcal infection OR gram-negative rod infection)

#3 (#1 AND #2)

#4 (Addresses[ptyp] OR Autobiography[ptyp] OR Bibliography[ptyp] OR Biography[ptyp] OR pubmed books[filter] OR Case Reports[ptyp] OR Congresses[ptyp] OR Consensus Development Conference[ptyp] OR Directory[ptyp] OR Duplicate Publication[ptyp] OR Editorial[ptyp] OR Systematic reviews OR Meta analysis OR Festschrift[ptyp] OR Guideline[ptyp] OR In Vitro[ptyp] OR Interview[ptyp] OR Lectures [ptyp] OR Legal Cases[ptyp] OR News[ptyp] OR Newspaper Article[ptyp] OR Personal Narratives [ptyp] OR Portraits[ptyp] OR Retracted Publication[ ptyp] OR Twin Study[ptyp] OR Video-Audio Media[ptyp])

#5 (#3 NOT #4)

1. Search strategy for Embase

(ultraviolet-C or UV light or pulsed xenon ultraviolet or health care disinfection and (nosocomial infection) or (disinfec*) AND 'ultraviolet':ti,ab,kw or “UV-C’; ti,ab,kw  or “health care infection”: ti,ab,kw

1. Search strategy for Scopus

#1 (ultraviolet-C) or (UV-C) or (pulsed xenon) OR TITLE-ABS-KEY (ultraviolet)

#2 (nosocomial) or (health care infection) or (clostridium difficile) or (vancomycin resistance) OR TITLE-ABS-KEY (health care infection)

#1 and #2
