# Supplementary material for: Effectiveness of ultraviolet-C disinfection systems for reduction of multi-drug resistant organism infections in healthcare settings: A systematic review and meta-analysis
Source: Epidemiol Infect. 2023 Aug 30;151:e149. doi: 10.1017/S0950268823001371 (PMC10540170; doi:10.1017/S0950268823001371)
Supplement: Sun et al. supplementary material 7 — Sun et al. supplementary material [file S0950268823001371sup007.docx]

**Supplementary table 2. Author’s judgements about study quality for studies with a before-after design with no control**

|  | McMullen (2021) | Abosi (2021) | Steele (2021) | Napolitano (2015) | Schaffzin (2020) |
| --- | --- | --- | --- | --- | --- |
| Was the study question or objective clearly stated? | Y | Y | Y | Y | Y |
| Were eligibility/selection criteria for the study population pre-specified and clearly described? | Y | Y | Y | Y | Y |
| Were the participants in the study representative of those who would be eligible for the test/service/intervention in the general or clinical population of interest? | Y | Y | Y | Y | Y |
| Were all eligible participants that met the pre-specified entry criteria enrolled? | Y | Y | Y | Y | N |
| Was the sample size sufficiently large to provide confidence in the findings? | Y | Y | Y | Y | Y |
| Was the test/service/intervention clearly described and delivered consistently across the study population? | Y | Y | Y | N | Y |
| Were the outcome measures pre-specified, clearly defined, valid, reliable, and assessed consistently across all study participants? | Y | N | Y | Y | N |
| Were the people assessing the outcomes blinded to the participants' exposures/interventions? | N | N | Y | N | Y |
| Was the loss to follow-up after baseline 20% or less? Were those lost to follow-up accounted for in the analysis? | N | N | Y | N | N |
| Did the statistical methods examine changes in outcome measures from before to after the intervention? Were statistical tests done that provided p values for the pre-to-post changes? | Y | Y | Y | Y | Y |
| Were outcome measures of interest taken multiple times before the intervention and multiple times after the intervention (i.e., did they use an interrupted time-series design)? | N | Y | N | N | Y |
| If the intervention was conducted at a group level (e.g., a whole hospital, a community, etc.) did the statistical analysis take into account the use of individual-level data to determine effects at the group level? | N | N | N | N | N |
| Overall risk of bias (Scores for each criterion added together and divided by 12; Yes=1; No=0); Low (75-100%); moderate (25-75%); high (0-25%) | Moderate | Moderate | Low | Moderate | Moderate |
